# Evaluation of Pharmacists’ Preferences and Barriers to Access Continuing Education: A Cross-Sectional Study in Lebanon

**DOI:** 10.1177/01632787221126500

**Published:** 2022-09-07

**Authors:** Georges Hatem, Lina Ismaiil, Sanaa Awada, Diana Ghanem, Roula Bou Assi, Mathijs Goossens

**Affiliations:** 1 Clinical and Epidemiological Research Laboratory, Faculty of pharmacy, 63572 Lebanese University, Lebanon; 2 Faculty of Medicine, 26706 University of Porto, Porto, Portugal; 3 Faculty of public Health, 214759 Lebanese University, Lebanon; 4 Center for Cancer Detection, 70493 Vrije Universiteit Brussel, Belgium

**Keywords:** continuing education, pharmacists, preferences, barriers, learning

## Abstract

The implementation of continuing education programs for pharmacists in Lebanon is emerging and needs to be further developed and strengthened to fill the gaps between knowledge acquisition and its application in the workplace. This study examined the perceptions of pharmacist preferences for and barriers to access programs. A crosssectional descriptive study was undertaken with a convenience sample of 142 pharmacists who were surveyed in their workplace. Almost 83.1% of pharmacists reported their day-to-day workplace experiences were the best way to learn. The high cost (50%) and time away from work (37.8%) were the main barriers to continuing education. Pharmacists reported a mean satisfaction of 5.8 (sd = 2.2)/10 with programs suggesting a need for routine needs assessments and adaptation of programs to better meet their learning needs.

## Introduction

Effective lifelong learning systems allow healthcare professionals to stay up-to-date with the latest medical developments, enhance their clinical knowledge and ensure awareness of the new challenges in society (Aldosari et al., 2020; Chan et al., 2021). The International Pharmaceutical Federation supports pharmaceutical stakeholders worldwide in providing pharmacists with access to continuing education (CE), motivating them to participate in the programs, and establishing quality assurance systems for the different programs (Hajj et al., 2022). There are increasing expectations for pharmacists to participate in CE (Gallegos et al., 2021; Sepp et al., 2021). Pharmacists who participate in CE to improve professional knowledge and skills have demonstrated higher efficiency and performance (Tofade et al., 2010).

The implementation of CE programs in Lebanon is in development and needs to be advanced and strengthened to meet the needs of pharmacists in all sub-sectors including community and hospital pharmacists, medical representatives and researchers (Hatem et al., 2021). The Order of Pharmacists in Lebanon (OPL) is pursuing the implementation of a law that makes CE mandatory (law number 190, November 2011) and is encouraging enrollment for pharmacists from all specialties. In December 2015, the OPL started providing programs to further develop the pharmacists’ profession and enhance patient outcomes considering the challenges and barriers previously reported (Sacre et al., 2019). The OPL organises conferences, congresses and online courses that are provided free of charge. Each program is awarded study credits. Every pharmacist needs to gather up to 15 credits per year; 10 credits can be obtained through online courses (Iskandar et al., 2018).

Meeting individual learning needs and preferences can support pharmacists in their current and future professional functions (Schindel et al., 2019). Previous research has suggested the importance of adapting pharmacist CE programs in Lebanon to their professional role given the differences in the preferences and barriers they face to access CE (Sacre et al., 2019). Moreover, the participation rates and perceptions towards CE vary between male and female pharmacists and different preferences have been reported previously (Hatem et al., 2021). Assessing the differences in pharmacist perceptions according to their professional role and sex is important and can provide additional information for CE providers. Evaluating satisfaction can help understand the effectiveness of the current programs. This study aimed to (i) investigate pharmacists’ preferences for and barriers to CE in Lebanon, (ii) how they are associated with sex and professional roles, and (iii) satisfaction with CE programs.

## Methods

### Study design and sample

A cross-sectional descriptive study was conducted. Data were collected over a period of 3 months (July-September 2017) through face-to-face interviews with three trained pharmacists to ensure quality and optimize response.

A non-probability sample was frequency matched to national government estimates of pharmacists’ sex and distribution in five distinct Lebanese governorates: Beirut, South, North, Beqaa, and Mount Lebanon (Supplemental Table A1, Appendix) (Alameddine et al., 2019). The required sample size was calculated according to calculations done on the “Epi-info” program that is based on the following equation

n=(Z1−α2)(1−p)/d2

where Z is a standard normal variate (Z1-α/2 = 1.96 at 95% confidence interval), P is the expected proportion of outcome in the population based on other studies, and d is the absolute accuracy or precision (8% marginal error). This resulted in a required sample size of 142 pharmacists.

Pharmacists were visited in their workplace at any time of the day either in community pharmacies or in hospitals (for pharmaceutical company representatives). Hospital and clinical pharmacists were excluded. The interviews were approximately 12–15 minutes long.

### Study instrument

The survey (Supplemental Appendix) was prepared through expert opinion and piloted with 15 pharmacists. Questions that lacked clarity or comprehensiveness were edited. The final interview consisted of close-ended questions and collected the general characteristics of the participants. This included sex, age, work location (hospital, community pharmacy) and professional role (manager, entry-level pharmacist), type and source of pharmacy certificate and duration of practice as well as their preferred learning methods and different barriers to accessing CE (see Table 1, multiple answers possible). Participants were asked to rank their satisfaction related to CE programs they had undertaken and whether the CE programs achieved their goal(s) and matched their learning needs.

**Table 1.** Pharmacists’ preferred methods of continuing education and reported barriers to continuing education by sex and job position*. Lebanon 2017.

		Sex			In a Pharmacy		In a Company		
Preferred Methods	N (%)	Male	Female	*p*-value	Manager	Entry-level	Manager	Entry-level	*p*-value
Day-to-day workplace experiences	118 (83.1%)	38 (71.7%)	80 (89.9%)	0.005	17 (58.6%)	54 (90%)	17 (85%)	24 (88.9%)	0.002
Group work	56 (39.4%)	14 (26.4%)	42 (47.2%)	0.014	12 (41.4%)	20 (33.3%)	14 (70%)	10 (37%)	0.036
Lectures	43 (30.3%)	23 (43.4%)	20 (22.5%)	0.009	11 (7.9%)	12 (20%)	6 (30%)	11 (40.7%)	0.155
Reading privately	56 (39.4%)	27 (50.9%)	29 (32.6%)	0.030	15 (51.7%)	35 (41.7%)	--	13 (48.1%)	0.001
Online courses	41 (28.9%)	18 (34%)	23 (25.8%)	0.302	12 (41.4%)	14 (23.3%)	6 (30%)	6 (22.2%)	0.294
Talking to colleagues	41 (28.9%)	15 (28.3%)	26 (29.2%)	0.908	3 (10.3%)	35 (58.3%)	3 (15%)	--	<0.001
Workshop	33 (23.2%)	8 (15.1%)	25 (28.1%)	0.076	7 (24.1%)	12 (20%)	6 (30%)	8 (29.6%)	0.654
Barriers to continuing education	N (%)	Male	Female	*p*-value	Manager	Entry-level	Manager	Entry-level	*p*-value
Time away from work	53 (37.8%)	21 (39.6%)	32 (36%)	0.662	20 (69%)	24 (40%)	3 (15%)	6 (22.2%)	<0.001
Cost	66 (48.6%)	19 (35.8%)	50 (56.2%)	0.019	15 (51.7%)	30 (50%)	14 (70%)	7 (25.9%)	0.025
External demands	34 (23.9%)	11 (20.8%)	23 (25.8%)	0.492	3 (10.3%)	18 (30%)	3 (15%)	10 (37%)	0.064
Lack of motivation	38 (32.4%)	17 (32.1%)	29 (32.6%)	0.950	12 (41.4%)	14 (23.3%)	6 (30%)	6 (22.2%	0.294
Past negative experience	27 (19%)	8 (15.1%)	19 (21.3%)	0.358	3 (10.3%)	14 (23.3%)	3 (15%)	7 (25.9%)	0.338
Work-life balance	73 (55.6%)	31 (62.3%)	42 (51.7%)	0.220	15 (51.7%)	27 (45%)	11 (55%)	20 (74.1%)	0.094

*N (%): Frequency (percentage) *Pharmacists were allowed to mark more than one answer.

### Statistical analysis

Data analyses were performed using SPSS (Statistical Package for Social Sciences) version 26. Descriptive statistics were used to report the general characteristics of respondents. Frequencies and percentages were used to describe all characteristics. Satisfaction scores were categorized into poor satisfaction (<5), moderate satisfaction (5–7), and high satisfaction (>7). Bivariate analyses were undertaken. The dependent variables were the methods preferred to access CE and the independent variables were sex and the professional role of pharmacists. Chi-square/Fisher exact test was used to test for differences in preferences and perceived barriers according to sex and professional role depending on the work location. A *p*-value less than 0.05 was considered statistically significant.

### Ethical considerations

This study did not require formal ethical review as it complied with the Lebanese University ethical committee criteria: data were anonymous and non-identifiable, data were not considered sensitive or confidential, the subject matter was limited to topics within the professional competence of the participants, storage of data followed university general data protection regulation guidelines, and full informed consent was obtained before each interview. Participants were informed their participation was voluntary and their anonymity was guaranteed in the study, the names of participants were not registered and they could withdraw their participation during the interview.

## Results

### General Characteristics of the participants

In total, 193 pharmacists were contacted and 142 (73.6%) agreed to participate. The sample distribution of place of work and sex was similar to the national database, although the sample overrepresented the pharmacists in Beirut. Most of the participants were less than 25 years of age (56.3%). Participants had different levels of education with the largest percentage achieving bachelor’s degrees in pharmacy (42.3%). They were mainly graduates of the Lebanese University (62.5%), the only public university with a faculty of pharmacy in Lebanon. The sample included community pharmacists and pharmacists working for pharmaceutical companies. The highest percentage accounted for those working in an entry-level position inside community pharmacies (44.1%) compared to almost 20% in the same position in companies (Supplemental Table A2, Appendix).

### Pharmacists’ preferred methods and barriers to CE and the association with sex and professional roles

Day-to-day workplace experiences (interaction with patients, colleagues and other health professionals) were pharmacists’ most preferred learning method (83.1%) followed by group work (e.g., small group discussions, role-playing exercises within a course) (39.4%), reading (39.4%) and attending lectures (30.3%). Sex was significantly associated with preference for learning methods: day-to-day experience (72% of males, 89.9% of females, *p* = 0.005); group work (73.6% of males, 52.8% of females, *p* = 0.014); lectures (43.4% of males, 22.5% of females, *p* = 0.009); reading (50.9% of males, 32.6% of females, *p* = 0.030). Moreover, 58% of managers in community pharmacies considered their day-to-day workplace experiences to be their preferred learning method compared to almost 90% of pharmacists working in an entry-level position in both companies and pharmacies (p-0.002). In addition, 70% of managers in community pharmacies considered group work as their preferred method to access CE compared to only 41.4% of managers in pharmaceutical companies (*p* = 0.036).

The main barrier reported by 55.6% of participants was work-life balance. Almost 38% of pharmacists considered time away from work to be their barrier to accessing CE and 48.6% reported the high cost of the programs as the main constraint. A significantly higher percentage of females (56.2%) considered the cost of the programs as their main barrier compared to 35.8% of males (*p* = 0.019). Taking time away from work was the main barrier was reported as the main barrier by 69% of managers in community pharmacies compared with 40% of those in entry-level positions (*p* < 0.001). Almost 52% of managers in community pharmacies reported that the cost of the programs limited their access to CE compared to only 26% of pharmacists in an entry-level position in companies (*p* = 0.025) (Table 1).

### Satisfaction with CE programs

Pharmacists reported a mean satisfaction score of 5.8±2.2 out of 10 distributed as follows: poor satisfaction (29.6%), moderate satisfaction (44.4%), and high satisfaction (26%). Furthermore, when asked whether CE programs provided by the OPL have accomplished their intended goals, only 28.9% agreed, despite 62% reporting that these programs matched their learning needs.

## Discussion

Overall, respondents said day-to-day workplace experiences and group work were their preferred methods of learning. A good work-life balance was the main barrier to CE. Sex and professional roles seemed to significantly affect both pharmacists’ preferences and perceived barriers. Moderate levels of satisfaction with the programs were reported.

Day-to-day workplace experiences were the most preferred method of CE, a finding consistent with that of other health professionals (Mlambo et al., 2021). This finding highlights the importance of including one-to-one learning methods, problem-solving, role plays, and case-based learning options in the programs to increase procedural skills effectiveness (Steenhof, 2020) to ensure CE is aligned with workplace learning needs. Men preferred group work and lectures more than women. This result may be explained by the additional tasks women have reducing their time flexibility to attend in-person CE programs (Croda & Grossbard, 2021). Furthermore, sex was significantly associated with choosing day-to-day experiences as the most preferred method: a higher percentage of women favoured it over traditional learning methods similar to previous research (Eksteen et al., 2018). Pharmacists working in entry-level positions had significantly higher preferences for day-to-day learning experiences, possibly related to their limited practical experiences. This emphasizes the importance of ensuring graduating pharmacists are taught in a curriculum that is integrated to place content in context, emphasizes critical thinking, and authentic problem-solving skills, and helps them retain and apply the skills and knowledge required of a practice-ready pharmacist (Wright et al., 2018). By contrast, managers in community pharmacies preferred group work compared to those working for pharmaceutical companies which may relate to the preferences of community pharmacists for formal learning with its proven effectiveness in enhancing participant engagement and changes in practice (Davis et al., 1999).

Work-life balance, the cost of programs and the difficulty obtaining time off work were the main barriers to CE, a finding similar to previous work in Lebanon (Saade et al., 2018). Females were more likely than males to mention the cost of CE as their main barrier, a finding consistent with other research (Chuang, 2015). Time away from work was reported to be the main barrier to access CE by more managers in community pharmacies in agreement with a study performed in the United Kingdom in 2014 (Ikenwilo & Skåtun, 2014). Moreover, pharmacists in managerial positions reported that the high cost of the programs limited their access to CE, a finding similar to a baseline survey conducted in Ethiopia in 2018 (Gelayee et al., 2018). In this study, almost 44% of pharmacists showed moderate satisfaction which can be related to the differences in preferences, interests and barriers faced (Hasan, 2009). Access to CE is important to increase job satisfaction and improve practice (Gustafsson et al., 2018). CE program providers can enhance pharmacists’ satisfaction and participation by providing different learning options.

Lebanese pharmacists appeared unfamiliar with CE opportunities; this study can provide national baseline data on the Lebanese overall healthcare sector, pharmacists’ perceptions, and the practices regarding CE programs. Additionally, it suggests including undergraduate pharmacy students in selected CE programs which may introduce CE to them early and establish the need for continuous learning throughout their careers.

The present study has limitations. Slightly over 25% of pharmacists contacted could not be interviewed. As the majority of community pharmacists in Lebanon are owned and managed by one pharmacist, their workload may have precluded participation in the study. Recall bias could have arisen given the limited time to complete the survey; however, interviewer bias was reduced by providing appropriate training to pharmacists collecting data from the sample.

## Conclusion

Pharmacists perceived several types of CE program options as important resources for their professional development with higher preferences for day-to-day workplace experiences. Different barriers were reported including work-life balance, high cost and time away from work. Taking into consideration the effectiveness of these programs in improving their knowledge, pharmacists’ needs should be routinely assessed and CE should be adapted accordingly. As CE develops in Lebanon, steps to increase self-motivation for learning and reduce costs may be necessary to ensure positive attitudes to self-learning in the pharmaceutical workplace.

## Supplemental Material

Supplemental Material - Evaluation of Pharmacists’ Preferences and Barriers to Access Continuing Education: A Cross-Sectional Study in Lebanon Click here for additional data file. Supplemental Material for Evaluation of Pharmacists’ Preferences and Barriers to Access Continuing Education: A Cross-Sectional Study in Lebanon by Georges Hatem, Lina Ismaiil, Sanaa Awada, Diana Ghanem, Roula Bou Assi, Mathijs Goossens in Evaluation & the Health Professions
