# Supplementary material for: Evaluation of Pharmacists’ Preferences and Barriers to Access Continuing Education: A Cross-Sectional Study in Lebanon
Source: Eval Health Prof. 2022 Sep 7;45(4):420–4. doi: 10.1177/01632787221126500 (PMC9630953; doi:10.1177/01632787221126500)
Supplement: Supplemental Material - Evaluation of Pharmacists’ Preferences and Barriers to Access Continuing Education: A Cross-Sectional Study in Lebanon [file sj-pdf-1-ehp-10.1177_01632787221126500.pdf]

Appendix

Supplementary Tables

Table 1: Distribution of the pharmacists between different governorates and according to their sex (Comparison between the study sample and the ministry of public health data in 2016).

	Frequency in the study sample	Percentage (%)	Ministry statistics in 2016	Percentage (%)
**Governorate**				
Beirut	39	27.5	1537	18.2
Mount Lebanon	69	48.6	4297	50.7
North	13	9.2	1011	11.9
South	11	7.7	746	8.8
Beqaa	10	7	876	10.4
**Sex**				
Male	53	37.3	3337	37.7
Female	89	62.7	5518	62.3

Table 2. General and job characteristics of the study participants. Lebanon 2017

		Frequency (%)
**Age** (Years)	Less than 25	80 (56.3%)
	25-35	30 (21.1%)
	35-45	21 (14.8%)
	More than 45	11 (7.8%)
**Type of certificate**	Pharmacy	60 (42.3%)
	PharmD	32 (22.5%)
	Master	39 (27.5%)
	PhD	11 (7.7%)
**Source of certificate**	Public University	85 (62.5%)
	Private University	35 (25.7%)
	Outside Lebanon	16 (11.8%)
**Position of Pharmacists**	Manager in a pharmacy	29 (21.3%)
	Manager in a company	20 (14.7%)
	Entry-level in a pharmacy	60 (44.1%)
	Entry-level in a company	27 (19.9%)
**Years of Practice**	Less than 5	97 (68.3%)
	5-9	25 (17.6%)
	10-14	5 (3.5%)
	>15	15 (10.6%)

Questionnaire in English

نرجو المشاركة في الإحصاء بالرد على الأسئله المطروحة مع تعهدنا على عدم إستعمال المعلومات المعطاة إلا فإطار الدراسة العلمية البحتة التي نجريها

Part 1: Demographic Data

	Demographic Data				
Gender	□ Male	□ Female			
Age	------				
District/City of work	□ Beirut	□ Bekaa	□ South	□ North	□ Mount Lebanon
Type of certificate	□ Pharmacy	□ PharmD	□ Masters	□ PhD	
Source of certificate	□ LU	□ Private	□ Outside Lebanon		
Period of practice	-----				

Part 2: Data on Perception regarding the effectiveness of CPD

What CPD have you done in the past 12 months? Please tick all that apply

CD/DVD learning	
Conference attendance	
Drug companies materials/ events	
E Learning module	
Online conference	

In what ways was the CPD successful/ worthwhile/ inspirational, or otherwise?

	**Strongly agree**	**Agree**	**Neutral/Don't know**	**Disagree**	**Strongly Disagree**
Change in attitude					
Change in practice					
Improved practical skills					
Knowledge acquisition					
Learner satisfaction					
Patient outcome					

I consider CPD to be:

	**Strongly agree**	**Agree**	**Neutral/Don't know**	**Disagree**	**Strongly Disagree**
A chore					
Bureaucratic					
Enjoyable					
Natural part of professional life					
Necessary for patient safety					
Necessary for career progression					
Rewarding					
Threatening					
Unnecessary					

Who should be responsible for the quality of CPD provision? Tick all that apply

Colleges/Faculties	
Deaneries	
Local providers	
Specialist societies	
Other (Please specify):	

Who should decide on the CPD curriculum?

Colleges	
Employers	
Governments	
Patients	
Self-directed	
Other (Please specify)	

<u>Part 3: Data on Self-learning and continuous education</u>

How do you learn best? (Tick all that apply)

Day-to-day workplace experience	
Group work	
Lecture	
Online courses	
Problem solving	
Reading privately	
Talking to colleagues	
Tonto (teaching one to one)	
Workshop	

What are your barriers to CPD learning? Tick all that apply

Availability of study leave	
Cost	
External demands	
Motivation	
Past negative experiences	
Work-life balance	

What best contributes to your CPD?

College conference	
OPL learning program	
Drug companies	
E-Learning	
Local provision	

Do you think that the continuous education program provided by the OPL has achieved its goals?

□ Yes □ No

Do the CPD programs you have done match your needs in learning?

□ Yes □ No

Rank your satisfaction regarding CPD programs done: (over 10)

- - - -

Thank you
